# The dark side of leadership: How ineffective training and poor ethics education trigger unethical behavior?

**DOI:** 10.3389/fpsyg.2022.1063735

**Published:** 2022-12-01

**Authors:** Abderrahmane Benlahcene, Oussama Saoula, Mathivannan Jaganathan, Abbas Ramdani, Nagwan Abdulwahab AlQershi

**Affiliations:** ^1^School of Government, Universiti Utara Malaysia, Sintok, Malaysia; ^2^School of Business Management, Universiti Utara Malaysia, Sintok, Malaysia; ^3^College of Arts, Sciences and Information Technology, University of Khorfakkan, Khor Fakkan, United Arab Emirates; ^4^Malaysian Graduate School of Entrepreneurship and Business, Universiti Malaysia Kelantan, Kota Bharu, Kelantan, Malaysia

**Keywords:** ethics training, ethics education, leadership, unethical leadership, public companies, Algeria

## Abstract

**Introduction:**

The challenge of restricting unethical behavior requires public companies to reinforce ethical practices among leaders through various instruments. Previous research suggests that the (un)ethical behavior of leaders can be influenced by many situational factors. This study aimed to investigate the influence of ethics training and education on unethical leadership behavior in the Algerian public companies.

**Methods:**

Data were collected through semi-structured interviews with 15 leaders from public companies in Algeria. Data analysis was facilitated using ATLAS. ti 8 qualitative analysis software.

**Results:**

The findings show that public companies in Algeria suffer from several issues related to leaders’ ethics training and education. The findings also indicate that some of these unethical leadership behaviors are the result of ineffective training programs and poor ethics education within public companies.

**Discussion:**

The absence or ineffectiveness of ethics training and education within and outside organizational settings has a detrimental impact on leaders’ ethical character. This study is the first to explore how public companies in Algeria engage in ethical training and leadership education. The different sectors of the Algerian business can use the findings as a point of reference to embed the appropriate ethical climate in their respective organizations.

## Introduction

The continuous challenges in the business world, coupled with exponential growth in many sectors, require companies to employ skilled and competent individuals. This is particularly true regarding the quality of company leaders. One way to ensure organizations’ long-term development is to implement dynamic training and development for members (Barbosa and Sousa, 2020). Training is an indispensable system to reinforce learning and improve job performance. The key objective is to create sustainable changes in cognition and behavior for organizational members to acquire the competencies needed to perform their roles (Salas et al., 2012). Leadership development initiatives and programs in organizations have a crucial influence on culture, productivity, growth, market share, and profits (Hurt and Homan, 2005). Moreover, ethical awareness and the skills that enable leaders in all organizational settings to deal with complex ethical decision-making processes are at the core of current scholarly discussions (Shakeel et al., 2019). Hence, training and educating public sector companies’ leaders to be able to address the increasing ethical implications of their decisions is as important as preparing them with technical and managerial skills.

The prevalent corruption and unethical practices in the public and private sectors have brought leadership ethics to the center of organizational studies (Romious et al., 2016; Holt et al., 2018). Thus, organizations have sought to restrict ethical deviations by instituting educational and training programs for leaders. Moreover, previous investigations have proved that organizations with higher ethical commitment engage in less earnings management, have better organizational performance and a higher market valuation, and achieve higher corporate financial performance (Ghazali, 2015). On the other hand, environments infiltrated with unethical behaviors have significant detrimental effects on the organization (Kirsten et al., 2017). Unethical leadership behaviors have a deleterious effect on employees and the entire organization (Brown et al., 2005; Erickson et al., 2007; O’Keefe et al., 2020). Specifically, unethical behavior has financial implications that directly contribute to corporate failure (Adeyanju, 2014). Therefore, the importance of leaders’ ethics training and education cannot be stressed enough. Ethical training is perceived as a decisive factor in decision making (Teixeira et al., 2018), assisting in clarifying ethical dilemmas (Chaplais et al., 2016), and creating an adequate space for organizational members to reflect on particular situations that occur during regular work in organizations (Pliscoff-Varas and Lagos-Machuca, 2021).

Organizations tailor different training and educational programs to cultivate good behavior among members. In line with this, various studies have established the positive influence of leadership training across different industries, outcomes, and settings (Hasson et al., 2016). Among these training programmes, ethics training is decisive in developing leaders’ ethical character (Beeri et al., 2013; Asencio, 2021). Ethical programs represent a set of processes and mechanisms by which organizations shape employees’ behavior, explaining both ethical and unethical forms of behavior within the organizational environment. In addition, ethical programs enable managers to prevent unethical behaviors. These programs have the potential to shape the organizational culture and establish a sound ethical atmosphere concerning business activities (Remišová et al., 2019). Furthermore, any effort geared toward the development of ethical leadership is critical for achieving sustainable business outcomes in the current complex and hyperconnected business world (Kvalnes and Øverenget, 2012; Turner et al., 2018). However, the development of leaders is not something that a company can do in the spur of the moment. The process takes time, close consideration, and assessment of both individuals and the organization (Hurt and Homan, 2005). Effective organizations offer personalized development and training practices that help leaders translate the vision, mission, and strategy of the organization into actions (Holt et al., 2018).

In light of the aforementioned, this study investigates the influence of ethics training and education on unethical leadership behavior within Algerian public companies. The current situation of leaders’ ethical performance and the ethical environment within public companies in Algeria is reflected in the ongoing issues of administrative corruption and unethical practices during the last three decades. One of the key factors contributing to the poor performance of the Algerian public sector organizations is widespread unethical practices among leaders and managers at the state level and different levels of the administration (Chama, 2019; Bennihi et al., 2021; Elsayed, 2021). Despite the importance of this issue and the proliferation of studies on leadership ethics in recent decades, research addressing leaders’ ethical and unethical behavior remains an unmapped field within the Algerian context (Benlahcene and Meddour, 2020). The literature provides minimal evidence of the role and influence of leadership ethics training and education in Algerian public companies. Several studies have proposed that formal training in the multifaceted components of leadership is essential and should begin in the early career stages. Nevertheless, to date, the number of effective and thorough leadership training opportunities is inadequate at any career level (Sonnino, 2016; Flaig et al., 2020; Osuagwu, 2022). According to Pliscoff-Varas and Lagos-Machuca (2021), studies on the issue of ethics training are limited, not only in the context of the public sector but also in the private sector (Pliscoff-Varas and Lagos-Machuca, 2021). Similarly, Bellou and Dimou (2022) suggested that the destructive side of leadership has received growing interest over the years but the eveidence from public sector companies and institutions continues to be limited (Bellou and Dimou, 2022). Despite the progress made in integration and synthesis, and the increase in leadership studies, researchers continue to stress a wide range of gaps in our understanding of leadership. One prominent gap is understanding the influence of leadership development and training on organizational performance (Seidle et al., 2016). According to Akanji et al. (2019), most of the established body of knowledge on organizational leadership originates from studies from the Western world, with a distinct lack of similar research in developing countries with different cultural and institutional systems. This signals the nature of leadership concepts and constructs in non-Western contexts (Akanji et al., 2019). Consequently, a better understanding of the role of ethics training and education in influencing unethical leadership behavior is needed to improve the quality of leaders within public organizations in developing countries with different cultural and organizational settings.

This study takes a qualitative approach to explore the influence of ethics training and education on unethical leadership behavior in Algerian public companies. Although the antecedents of unethical leadership behavior are entangled in various organizational, social, and cultural factors (Ünal et al., 2012; Lašáková and Remišová, 2017; Braun et al., 2018), this study focuses on the potential role of ethics training and education. The study seeks to expand the literature on the antecedents of unethical leadership behavior and unethical leadership broadly by exploring how ineffective training programs and poor ethics education trigger unethical behavior among leaders. In doing so, this study investigates the effectiveness, content, and impact of leadership development programs on leaders’ ethical conduct from the perspective and experience of individuals holding leadership positions in various public companies. For this study, the term leadership development describes all forms of training and education programs intended to improve leaders’ ethical performance within Algerian public companies.

## Materials and methods

An exploratory qualitative approach was adopted to investigate the influence of ethics training and education on unethical leadership behavior. Empirical studies on the different factors affecting leadership behavior in Algeria are scarce. Thus, using an exploratory qualitative approach helps uncover various unmapped issues related to unethical leadership behavior. This design is considered useful in examining issues and phenomena of a behavioral nature (Veal, 2005), and it is especially valuable for scrutinizing sensitive or personal issues (Creswell and Poth, 2016).

The target population comprised top managers and leaders from four Algerian public companies. The term “leader” here denotes individuals who are occupying or have occupied a formal leadership position, such as managers, supervisors, heads of departments, and directors. Purposive sampling was used to acquire reliable first-hand information when selecting the respondents. As noted by Guest et al. (2006), when the objective of the research is to explore or describe perceptions, behaviors, or shared beliefs among a relatively homogeneous group of respondents, a sample of 12 respondents will be satisfactory for the study. Nevertheless, researchers in this type of qualitative study should be flexible and field oriented. In addition, following Clarke and Braun (2018) conceptualization, the researchers decided that data saturation was reached in 15 interviews. Therefore, the interviewees numbered 15 leaders and top managers, including one female leader, with a mean age of 46.86 years. The primary criteria for selecting the participants were their years of experience, willingness to participate in the study, and role or position in a public company. The researchers purposefully selected participants that can best provide an understanding of the phenomenon under examination. The chosen participants that took part in the current study are leaders within Algerian public companies. Therefore, their knowledge, perspectives, and experiences are important in understanding the issue of the influence of ineffective ethics training and poor ethics education on unethical leadership behavior. Further details are provided in Table 1.

**Table 1:** Demographic data.

Respondents	Position	Years of company employment	Age	Gender
A1	HOD, HR	12	43	Male
A2	HOD, Finance	5	41	Male
A3	Former HOD, Finance	23	61	Male
A4	Head of Division, Environment Protection	/	46	Male
A5	New HOD, Administration and Logistics	5	32	Male
A6	Head of Sub-Department, BD	7	42	Male
A7	Chief of Services, TS	17	54	Male
A8	Supervisor of Department, Procurements	10/11	46	Male
A9	HOD, HR	21	50	Female
A10	HOD, Manufacturing	19	50	Male
A11	HOD, Finance and Accounting	12	42	Male
A12	HOD, Maintenance and Transportation	13	42	Male
A13	HOD, Operations	18	45	Male
A14	Chief of District	30	49	Male
A15	Former Deputy General Director	35	60	Male

HOD, Head of Department; HR, human resources; TS, transportation services; BD, business development.

Semi-structured interviews were conducted to collect the relevant data. The researchers developed the interview questions based on the objective of the study. The questions were constructed in an open-ended form to elicit relevant and rich data and reflect participants’ experiences and perspectives in their social world (Creswell and Creswell, 2017). The interview guidelines included opening remarks to explain the study’s objective to the participants, key concepts, and the confidentiality of the information being collected. The interviews were face-to-face, and the average duration of each session ranged from 30 to 90 min, depending on the respondents’ willingness to add further information. The researchers took notes to complement the voice recording device. Given the sensitivity of the issue under investigation, interviewees were informed that they were free to ask questions before the interview session began to clarify any concerns regarding the nature of the study. Although most respondents were slightly anxious about participating in the study, they were willing to participate after understanding the study’s objective, purpose, and nature.

To interpret the interviewees’ perspectives, the researchers adopted Braun and Clarke (2006) thematic analysis, a process for identifying, analyzing, and reporting themes within the data. The step-by-step guide suggested by Braun and Clarke (2006) was as follows: (1) transcribing, organizing, and reading the data thoroughly; (2) after reading and rereading the transcribed data, the researchers reflected and wrote down the initial codes and notes; (3) searching and forming themes; (4) reviewing and matching the themes to the general nature of the data; (5) defining, naming, and renaming themes; and (6) producing the thematic analysis’s final report to provide a nuanced and detailed account of each theme. The process of transcribing, analyzing, and reporting was facilitated by the qualitative analysis software ATLAS.ti 8, which was used to organize the data and present the transcribed content as consistent and logical structures.

## Results

**Figure 1:**
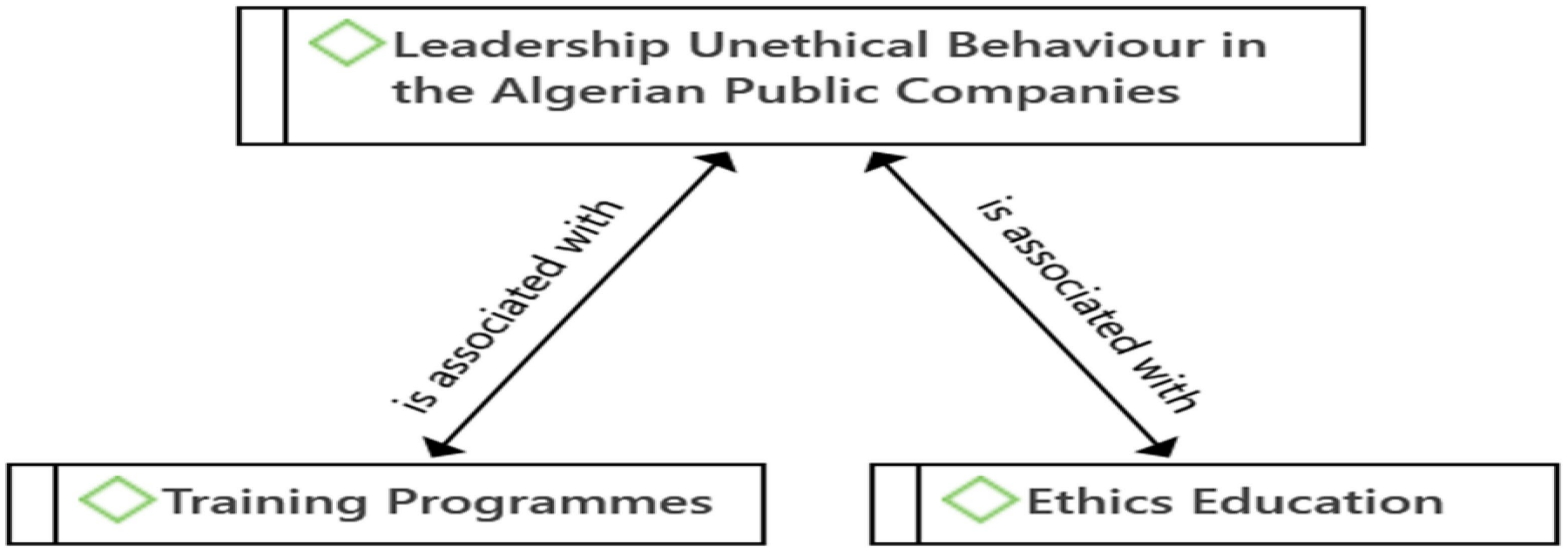
Leadership unethical behavior in the Algerian public companies.

What is the influence of ethics training and education on (un)ethical leadership behavior in Algerian public companies? Two themes related to this question emerged from analyzing the collected data, as shown in Figure 1. The first reported theme was ethics education, which included nurturing leaders’ ethical values in educational institutions, families, and communities. This theme encompasses the impact of ethics education on the ethical character of leaders before their recruitment to their respective companies.

The second theme is training programs that address the ineffectiveness and inadequate focus on building leaders’ ethical character in Algeria’s public companies. In response to ethics training, most respondents reported their perceptions and experience of the lack of ethical and managerial training initiatives and programs to establish and improve leaders’ ethical character within public companies in Algeria.

The perspectives and views of the respondents in this study suggest that there is inadequate ethics training and education for leaders. As expressed by the respondents, this situation has deprived leaders of proper ethical development and contributes to unethical behaviors within public companies. Moreover, most of the respondents opined that poor ethics education and the lack of training contributed to incompetence, unethical practices, and lack of skills that allow leaders to exhibit good organizational behavior. The reported themes and the respondents’ perspectives are presented in the following sections.

### Ethics education

The theme of ethics education represents respondents’ experiences and perspectives on educating and nurturing leaders with the right ethical values, especially concerning the critical role of ethics education at an early stage. The respondents stated that companies alone could not instil the right ethical values into the character of organizational members. This task requires the collaboration of educational institutions, society, and families to prepare future leaders who can perform their organizational duties ethically. Based on respondents’ perspectives and experiences, ethics education in the Algerian context is of poor quality and lacks effectiveness in forming ethical leadership behavior. Supporting the above view, Respondent A12 states:

*“Organisations, laws, or regulations cannot alone prepare or make ethical people, and this task needs more involvement in the early stages of education. If we want to have ethical individuals or leaders, we must think of ethical values before these leaders come to the organisation. The unethical leader will bypass the laws and ethical guidelines to get what he wants. So basically, if you have leaders with a strong sense of responsibility and ethical awareness, you will have a prosperous organisation*” (Respondent A12).

It is axiomatic that one of the essential steps in any business venture or organization’s success is to acquire well-educated and well-qualified individuals. The appointment of individuals with appropriate skills and values is important for organizations facing the compound challenges of the current business world. Accordingly, educational institutions, families, and other community entities are vital in forming individuals with a good character. However, the views echoed by the participants in this study imply that educational institutions and other social entities in Algeria do not play a pivotal role in preparing and nurturing future leaders with the appropriate ethical values. Furthermore, the struggle to prevent misconduct and unethical practices within organizational settings should begin at an early stage by equipping and nurturing individuals with ethical values. Responding to this issue, Respondent A3 demonstrated that organizational leaders should be educated in the right ethical values before their professional life.

“*Organisations cannot create competent and ethical leaders in an unhealthy environment or in an unethical environment. If we want to have good leaders in terms of ethics or competencies, this must happen before the person joins the organisation. In other words, it is a process and this process of making good leaders starts from the family, educational system, and society*” (Respondent A3).

Likewise, Respondent A13 considered ethics education to play a decisive role in shaping the ethical character of leaders.

“*I believe that the early ethical education and the ethical values which were adopted by the individual will lately determine what kind of leader he will be; that will decide if he is going to be an ethical or unethical leader and what to expect from him as an individual; this has a great effect on the moral conduct of leaders in Algeria”* (Respondent A13).

Despite differing views on the methods, models, and goals of ethics teaching, there is a general agreement that ethics can be taught. The usefulness and effectiveness of ethics education in improving ethical awareness, reasoning, and knowledge have been well established (Avci, 2017). In this study, the respondents’ shared view was that organizational rules and guidelines were insufficient to instil and promote good ethical behavior among leaders. This task requires active involvement of educational institutions and other social entities. The respondents’ statements suggest that the early stages of ethics education of prospective leaders are most likely to play a significant role in forming and predicting their ethical character in later stages. It is not easy to alter the ethical character of leaders once they are recruited as members of the organization.

### Training Programs

In this theme, in their accounts of leadership training programmes, the respondents were unanimous in that training programs, workshops, learning sessions, and policies within Algerian public companies were ineffective in molding leaders’ ethical character. They also indicated that Algerian public companies neglected the importance of training to improve leaders’ ethical performance. Their views suggest an apparent lack of investment in training and development, both in technical and ethical skills. Referring to the issue of training, one respondent stated the following:

*“There are programmes for development and training for leaders and employees as well, but the problem is that it is not implemented well, or the quality of these programmes is not really good. There is a huge lack of training and development regarding all types of training in the Algerian public sector. Human resource is the weakest point in the Algerian state-owned companies; we need more qualified, competent, and well-trained leaders and followers”* (Respondent A13).

Respondent A12 reported a lack of training and development programs for leaders in Algerian public companies.

*“I would say that there is a lack in terms of training, in terms of management and leadership programmes, and career development, workshops which focus on important ethical issues and other technical issues”* (Respondent A12).

Organizations are prone to corruption and unethical practices if they do not apply necessary measures. Regular training of organizational members is one of the most effective tools for restraining corrupt practices. Training is considered useful in minimizing organizational members’ possibility of becoming involved in unethical practices (Hauser, 2019). Hence, it is recommended that organizations implement ethics programs to reduce and prevent unethical practices (Kaptein, 2015; Hauser, 2020). However, participants’ responses suggest that public companies’ leaders require more training programs that can shape individuals’ ethical characteristics. The respondents also lamented the quality of existing training programs. For example, respondent A11 stated:

*“In order to equip our leaders with the right values for a better future we need more training and developmental programmes; actually, there are training programmes in the Algerian public organisation, but the quality of these programmes is not up to the expectations and challenges of this sector. They spend a lot on useless programmes!”* (Respondent A11).

Respondent A14 developed this position:

*“We need more training for leaders, we also need to create programmes where leaders can learn from others whether from Algeria or foreigners, and to gain new skills and new perspectives”* (Respondent A14).

Respondent A10 also claimed that:

*“During the last decade, there was a good improvement in terms of leaders’ and managers’ qualifications and skills. Yet, we are still suffering from the same ethical problems until this day. This is due to the lack of training regarding the importance of leadership and management ethics in public organisations”* (Respondent A10).

The respondents’ statements above indicate that the current quality of leadership training programs in Algerian public companies is not adequately designed to address the ethical dimensions of leaders’ roles at the organizational level. The purpose of training is to generate sustainable change in individuals’ cognition and behavior to develop the necessary competencies to perform their roles effectively. However, as seen in the respondents’ statements, training programs in Algerian public companies are not well-designed to positively change leaders’ behaviors.

## Discussion

Despite the practical importance of this issue, systematic studies on ethical and unethical leadership across sectors and cultures are rare (Eisenbeiß and Brodbeck, 2014). Research also points to the scarcity of empirical studies on the causes and consequences of unethical leadership behavior in public organizations (Hassan, 2019). This is particularly true in the context of Algerian public companies and other African countries. This exploratory study investigated the influence of ethics training and education on unethical leadership behavior based on leaders’ experiences and perspectives to fill this knowledge void. This study was set out to explore the link between ethics training and education on the one hand and unethical leadership practices in Algerian public companies on the other. Broadly, the current study was conducted to partially address the existing knowledge gap regarding unethical leadership behavior in Algerian public companies. The importance of exploring leaders’ ethical aspects stems from their decisive role in guiding organizational members toward the organization’s objectives (Eisenbeiß and Brodbeck, 2014). One of the critical responsibilities of good leaders is to guarantee that organizational activities are conducted ethically (Haq, 2011). Thus, leaders’ ethical deviance has a wider impact on the ethical culture of organizations and results in negative consequences for organizational performance.

Ethics training and education might not be the ultimate solution to leaders’ unethical behavior, yet it is an indispensable factor that organizations cannot neglect, especially in organizational environments where corruption and misconduct are common. According to Kim (2021), using ethics programs is important and effective in reducing incidents of unethical conduct among organizational members within public companies (Kim, 2021). Moreover, leadership training and education are necessary components of organizational activities in the current complex and dynamic business environments (Berkovich and Eyal, 2020). Previous studies have shown that unethical behaviors are less frequent in organizations with ethics programs than in those without them (Kaptein, 2015). Previous research also suggests that many factors shape leaders’ ethical conduct; among these factors, ethics programs and education play a crucial role in forming leaders’ ethical behavior at the organizational level (Schwartz, 2013).

Most organizations pay more attention to ethics training because of its significance in enhancing organizational performance (Harun et al., 2019). In line with this, a pressing issue in Algerian public companies is widespread unethical practices among leaders and top managers (Benlahcene and Meddour, 2020). Algerian public companies have long suffered the consequences of unethical leadership behaviors. Investing in the development of effective and ethical leaders is one way to overcome these challenges within these companies. This situation requires companies to steer their efforts towards developing and improving the quality of leaders to minimize the destructive consequences of unethical leadership activities on organizational performance.

Although this research is small-scale and exploratory, two critical insights can be drawn from the findings. First, although many studies have suggested that training programs positively influence leaders’ performance within organizational settings (Hasson et al., 2016; Riivari and Lämsä, 2019; Zhu et al., 2019), the findings of this study suggest that there is a lack of effective training programs for leaders within Algerian companies. Moreover, the respondents linked leaders’ ethical failures to a lack of such training programs. Trevino (1992) posited that organizational members’ ethical reasoning skills can be improved through effective training programs. This can be achieved by designing programs that contain practices in situational behaviors or moral role-taking, case studies, and group discussions to develop managers’ ability to deal with composite moral issues (Zhu et al., 2019). Leaders can be developed or, more accurately, they can learn the behavioral habits of effective leaders. They can change in desired ways, but not without effort or intent. By extension, teams, organizations, communities, and even countries can change in the desired ways, but without purposeful desire, the changes may be slow or result in unwanted consequences (Boyatzis, 2008). Likewise, ethical training programs based on sound principles and tools can effectively prepare business leaders and others for the dilemmas they encounter in their everyday work settings (Kvalnes and Øverenget, 2012).

Previous studies have suggested that ethics training programs must be provided to organizational members to develop their understanding of ethical decision-making and enhance their awareness of ethical values to build their moral reasoning (Valentine and Godkin, 2016). However, based on the analysis of the respondents’ perspectives, it appears that the absence of effective and well-structured ethics training programs for leaders negatively influences their ethical character and makes them more prone to ethical failure. Training is a valuable tool that can assist organizations in improving the ethical character of leaders. Kish-Gephart et al. (2010) suggest that adults’ moral development is generally stable, although it can continue to improve through training, practice, and learning.

Second, one of the most unexpected findings was the respondents’ emphasis on the role of leaders’ ethical education before their professional life. In addition to ineffective ethics training within public companies, the analysis of respondents’ perspectives suggested that early ethical development is decisive in forming individual leaders’ values, attitudes, and behaviors. This was evident in most of the respondents’ answers, which stressed the need for families, educational institutions, and society to prepare individuals with good ethical character. Leadership development includes all forms of growth and stages of development during the life cycle that encourage, assist, and promote the expansion and improvement of the experience and knowledge necessary to enhance the performance of leader (Packard and Jones, 2015).

Respondents reported that a significant part of leaders’ ethical development occurs before their recruitment into the organization. These findings suggest that early ethical development plays a decisive role in shaping and determining the ethical character of organizational leaders. The respondents echoed that the task of ethics training and education goes beyond the organizational environment to include the education system, families, and other entities within society. This study’s analysis suggests that social and cultural environments play a significant role in shaping leaders’ values, norms, and ethical awareness. In line with this, Day et al. (2014) stated that leadership development tends to begin at an early age and is partially influenced by parental modelling. It includes developing and applying a wide range of skills (e.g., creativity, intelligence, and wisdom), and is shaped by factors such as personality and relationships (Day et al., 2014).

From the findings of this study, it can be argued that the ethical development process of leadership is not the sole responsibility of organizational training and education. What is evident in the views and perspectives echoed by leaders of Algerian public companies is that the social environment (families and educational institutions) is the first platform where future leaders can be trained and educated in good ethical behavior. At the same time, the role of organizations is to establish and develop those values ingrained in leaders’ character to prepare them for the professional world. Moreover, organizations often draw from the recruitment pool without considering the ethical character of leaders. Therefore, one way to improve the quality of individual leaders in organizational settings is to ensure that different social and educational institutions play a role in embedding good ethical values into future leaders’ character.

## Conclusion

This study called into question the role of ethics training and education in predicting unethical leadership behavior in Algerian public companies. It sheds new light on a practical issue that lacked previous empirical investigations in the Algerian context by using an exploratory qualitative design that proved valuable in unveiling unmapped organizational issues. The findings of this study show that the absence or ineffectiveness of ethics training and education within and outside organizational settings has a detrimental impact on leaders’ ethical character. This implies that factors affecting leaders’ ethical development extend beyond organizational settings to include families, educational institutions, and wider social environments.

The findings of this study suggest that leaders from Algerian public companies perceive that organizations alone cannot instil good ethical values into the character of leaders. This task requires comprehensive collaboration between organizations and educational institutions, business schools, and families. The ethical behavior of leaders is crucial in shaping organizational culture and exerting a positive influence on followers. Thus, it is essential to investigate the various factors that might induce leaders to go astray and become involved in practices that are harmful to organizations and followers. The dearth of studies on organizational leadership in the Algerian context, especially in public companies, deprives this sector of a significant opportunity to optimize the quality of its leaders.

The findings of this study have significant implications for ethical development of leaders and the struggle against unethical practices within Algerian public companies and companies in other African countries. First, the findings offer important insights that support the assumption that ethics training programs positively influence leaders’ ethical character (Holt et al., 2018; Turner et al., 2018; Remišová et al., 2019). The findings propose that the absence of effective ethics training programs within organizational settings has a negative impact on leaders’ ethical performance. Second, the findings demonstrate that ethical education and development of leaders must occur at an early stage. Educational institutions and families play an essential role in shaping leaders’ ethical character. Third, these findings provide valuable insights for policymakers and organizational leaders seeking to promote and enhance the ethical performance of public companies’ leaders. These findings have important implications for education institutions. Business schools must include professional and normative ethics in their curricula. This can help familiarize future leaders with the various ethical obligations of the professional domain.

The current study had several limitations. The first concerns the sensitivity of unethical leadership behavior. Hence, it is possible that respondents were cautious when expressing their views on the issue within public companies. Thus, genuine views of unethical behavior cannot be taken for granted. The second limitation relates to the generalizability of the findings, as similar studies in other contexts may generate different results. The peculiarities of Algerian public companies, shaped by different historical, cultural, and structural features, might have geared the current study’s findings. Another limitation of this study is that female leaders were under-represented because of the dominance of male leaders within Algerian public companies. Despite these limitations, this study, by its exploratory nature, offers an in-depth examination of the link between ethics training and education and unethical leadership behavior in Algerian public companies. Therefore, further research should examine potential social and organizational antecedents of unethical leadership behavior in public companies, especially in the African context, where the rule of law is fragile and corruption ratios are high. Future studies should also investigate the implications of unethical leadership behavior on employees and public companies’ performance.

## Data availability statement

The raw data supporting the conclusions of this article will be made available by the authors, without undue reservation.

## Ethics statement

The studies involving human participants were reviewed and approved by the SoG, Universiti Utara Malaysia. Written informed consent for participation was not required for this study in accordance with the national legislation and the institutional requirements.

## Author contributions

AB contributed to the design and implementation of the research, analysis of the results, and writing of the manuscript. OS took the lead in writing the manuscript. MJ provided critical feedback and helped to shape the research and analysis. AR and NA contributed to the discussion of the results and the final manuscript. All authors contributed to the article and approved the submitted version.

## Conflict of interest

The authors declare that the research was conducted in the absence of any commercial or financial relationships that could be construed as a potential conflict of interest.

## Publisher’s note

All claims expressed in this article are solely those of the authors and do not necessarily represent those of their affiliated organizations, or those of the publisher, the editors and the reviewers. Any product that may be evaluated in this article, or claim that may be made by its manufacturer, is not guaranteed or endorsed by the publisher.
